# The Role of Glial Cells in the Pathophysiology of Epilepsy

**DOI:** 10.3390/cells14020094

**Published:** 2025-01-10

**Authors:** Filiz Onat, My Andersson, Nihan Çarçak

**Affiliations:** 1 Department of Medical Pharmacology, School of Medicine, Acibadem Mehmet Ali Aydinlar University, 34684 Istanbul, Türkiye; 2 Institute of Health Sciences, Department of Neuroscience, Acibadem Mehmet Ali Aydinlar University, 34684 Istanbul, Türkiye; 3 Department of Experimental Medicine, Faculty of Medicine, Lund University, 221 00 Lund, Sweden; my.andersson@med.lu.se; 4 Department of Pharmacology, Faculty of Pharmacy, Istanbul University, 34452 Istanbul, Türkiye

**Keywords:** astrocyte, microglia, oligodendrocyte, penta-partite synapse, seizure, experimental model, epileptogenesis

## Abstract

Epilepsy is a chronic neurological disorder marked by recurrent seizures, significantly impacting individuals worldwide. Current treatments are often ineffective for a third of patients and can cause severe side effects, necessitating new therapeutic approaches. Glial cells, particularly astrocytes, microglia, and oligodendrocytes, are emerging as crucial targets in epilepsy management. Astrocytes regulate neuronal homeostasis, excitability, and synaptic plasticity, playing key roles in maintaining the blood–brain barrier (BBB) and mediating neuroinflammatory responses. Dysregulated astrocyte functions, such as reactive astrogliosis, can lead to abnormal neuronal activity and seizure generation. They release gliotransmitters, cytokines, and chemokines that may exacerbate or mitigate seizures. Microglia, the innate immune cells of the CNS, contribute to neuroinflammation, glutamate excitotoxicity, and the balance between excitatory and inhibitory neurotransmission, underscoring their dual role in seizure promotion and protection. Meanwhile, oligodendrocytes, primarily involved in myelination, also modulate axonal excitability and contribute to the neuron–glia network underlying seizure pathogenesis. Understanding the dynamic interactions of glial cells with neurons provides promising avenues for novel epilepsy therapies. Targeting these cells may lead to improved seizure control and better clinical outcomes, offering hope for patients with refractory epilepsy.

## 1. Introduction

Epilepsy as a chronic neurological disorder is characterized by a persistent predisposition to generate recurrent and spontaneous seizures. An epileptic seizure is a transient occurrence of signs and/or symptoms due to abnormal excessive or synchronous neuronal and non-neuronal activity in the brain. According to the classification proposed by the International League Against Epilepsy (ILAE) in 2017, epileptic seizures are divided into three main groups: focal onset, generalized onset, and unknown onset [1,2]. Additionally, epilepsy classification based on predominant seizure type can be focal epilepsy, generalized epilepsy, combined generalized and focal epilepsy, or unknown [1,3]. In focal epilepsy, seizures remain localized to specific parts of the brain, while generalized epilepsy involves both hemispheres. Generalized epilepsy corresponds to a broad group of epilepsies with generalized seizures and a presumed genetic etiology. The ILAE defines six etiological categories: structural, metabolic, immune, infectious, genetic, and unknown [2,3].

Epilepsy with neurobiological, cognitive, psychological, social, and economic consequences affects approximately 50 million people of all ages worldwide, playing a role in worsening the global disease burden. As with all diseases, the goal in epilepsy is to achieve a complete cure, which means a seizure-free status. There are many challenges and struggles to cope with in epilepsy. Foremost among these is ensuring that anti-seizure drugs control epileptic seizures without achieving a cure. In other words, these drugs act directly on existing seizures, providing symptomatic treatment. Until recent years, the drugs used were known as anti-epileptic drugs; however, considering increasing knowledge, it has been understood that the existing treatments are far from providing a cure, and therefore they have been redefined as anti-seizure agents. Meanwhile, anti-epileptic or anti-epileptogenic therapy aims to counteract the progress of epileptogenesis and epilepsy. Epileptogenesis refers to the development of tissue capable of generating spontaneous seizures, resulting in (a) the development of an epileptic condition and/or (b) progression of epilepsy after it is established [4]. Moreover, the entire process of epileptogenesis seems to be modulated by genetic and/or acquired factors. In 30–40% of epileptic patients, epileptogenesis is initiated by structural initial insult such as traumatic brain injury, infection, intracranial aneurysms, or stroke [5]. In addition to the insults triggering the onset and progression of epileptogenesis, a significant majority of patients also have a genetic predisposition, which increases the likelihood of epilepsy development. Thus, the factors triggering epileptogenesis include not only an insult like trauma or infection but also a genetic predisposition/genetic factors to develop epilepsy. On the other hand, anti-epileptogenesis is a process that counteracts the effects of epileptogenesis, including prevention, seizure modification, and cure. Therefore, there is a need to discover new drugs, especially those that are effective against epilepsy, along with disease-modifying therapies that aim to provide modulatory effects on the underlying epileptic condition [6].

Another challenge in treating epilepsy is the inadequate control of epileptic seizures in patients despite receiving appropriate anti-seizure drugs. This difficulty, affecting approximately one-third of epileptic patients, is termed pharmacoresistant epilepsy, characterized by ongoing seizure activity despite the use of suitable anti-seizure medications. Additionally, the adverse effects of these drugs may reduce patient compliance, while toxic reactions can elevate the risks of mortality and other epilepsy-related health issues.

Taken together, there is a need for the discovery of novel antiepileptic drugs, particularly those effective on epileptogenesis, as well as disease-modifying therapeutics aiming at sustained modulatory effects on the underlying epileptic state.

**Figure 1:**
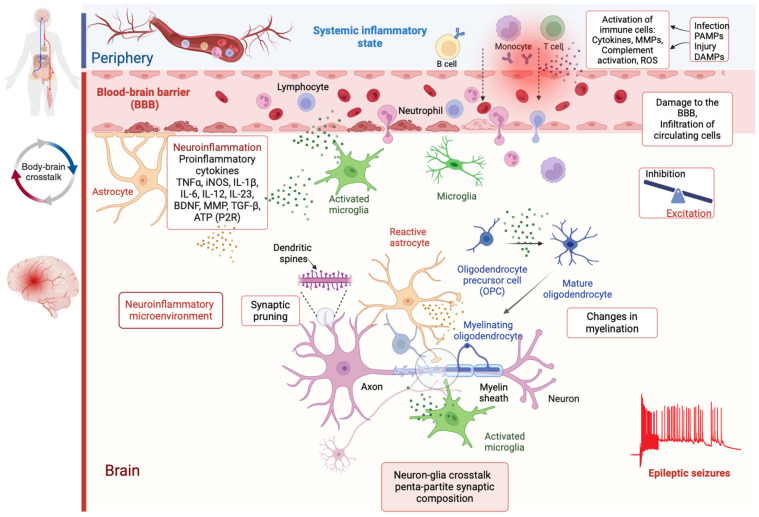
Interplay between systemic inflammation, neuroinflammation, and neuron–glia crosstalk in epilepsy. In a healthy brain, the primary cell types include neurons, astrocytes, microglia, oligodendrocytes, and oligodendrocyte precursor cells (OPCs). They are now recognized as essential synaptic partners forming the penta-partite synaptic composition. Neurons connect via long axonal processes and form synapses to communicate. Astrocytes interact with blood vessels to form the blood–brain barrier (BBB) and maintain neuronal synapses. Microglia, the innate immune cells of the CNS, phagocytose apoptotic cells and prune inactive synapses. Oligodendrocytes support axons by forming the myelin sheath that insulates axons, allowing for rapid conduction of action potential in the CNS. Various factors that contribute to the activation of immune cells lead to the release of cytokines, matrix metalloproteinases (MMPs), oxidative stress (reactive oxygen species—ROS), and complement activation. This systemic inflammatory response alters the BBB integrity/permeability, facilitates the infiltration of peripheral immune cells into the CNS, and activates microglia and astrocytes, further amplifying inflammation and BBB leakage in the CNS. Dotted arrows indicate the infiltration of circulating cells into the brain. B cells that cross the BBB can produce brain-reactive antibodies that can interfere with presynaptic and postsynaptic proteins and activate an autoimmune reaction within the CNS. Reactive astrocytes and activated microglia initiate an inflammatory cascade, releasing cytokines, chemokines, inflammatory mediators, and ROS, which can lead to excitotoxicity, oxidative stress, demyelination, increase in synaptic pruning, and apoptosis. Reactive glia releases extracellular matrix remodeling enzymes such as MMPs, neurotrophic factors such as brain-derived neurotrophic factor (BDNF), growth factors such as transforming growth factor-β (TGFβ) and cytokines that modulate the expression and function of receptors, transporters, ion channels, enzymes, other synaptic proteins, and molecules implicated in excitatory and inhibitory neurotransmission. MMPs can aggravate BBB impairment by damaging the tight junction proteins and promote infiltration of immune cells and serum proteins such as albumine in the brain. As a result, network hyperexcitability and seizures are promoted in this neuroinflammatory microenvironment. Created in BioRender 2024. (https://BioRender.com/t81x673, accessed on 10 November 2024).

Synaptic transmission is no longer considered unique to neurons, occurring only between pre- and postsynaptic neurons, but also involves mutual crosstalk between glial cells and neurons in regulating brain functions. Therefore, glial cells, primarily classified into astrocytes, microglia, oligodendrocytes, and oligodendrocyte precursor cells (OPCs), namely NG2 glia, are now recognized as essential synaptic partners [7]. OPCs are included in the penta-partite synaptic composition alongside neurons, astrocytes, microglia, and oligodendrocytes because they have unique and active roles in the neural network that go beyond their identity as precursor cells [8]. Unlike other precursors, such as those that give rise to astrocytes or microglia, OPCs demonstrate direct interaction with neurons and influence synaptic activity. These unique properties of OPCs justify their inclusion as a distinct and active component of the neural network and synaptic composition [8,9]. Over the last two decades, the concept of “tripartite synapse” has gained significant attention, highlighting astrocytes as essential partners of neurons in regulating synaptic activity [10,11]. However, it is becoming more evident that besides astrocytes, microglia and oligodendrocytes also play vital roles in neuronal structure and function, actively participating in synaptic transmission. This expanded view is now referred to as the “penta-partite synaptic composition” (Figure 1). In this model, each component—including neurons, astrocytes, microglia, oligodendrocytes, and OPCs—signals and communicates with each other to regulate the complex structural and functional microenvironment of the CNS [12]. This integrated composition highlights the collaborative nature of brain cell interactions in modulating synaptic transmission, maintaining neural networks, and influencing both physiological and pathological conditions, including epilepsy.

This review addresses the multifaceted roles of glial cells in epilepsy pathophysiology, highlighting their dynamic interactions with neurons and their contributions to seizure generation and modulation in both focal and generalized convulsive and non-convulsive epilepsies. Considering recent findings, we focus on the specific roles that astrocytes, microglia, and oligodendrocytes play in epileptogenesis with a deeper understanding of neuron–glia crosstalk. We hope that linking this evidence will provide deeper insights into the mechanisms of epileptogenesis and identify new therapeutic targets for anti-epileptogenesis.

## 2. Glial Contributions to Pathophysiology of Epilepsy

### 2.1. Astrocytes

Astrocytes are a heterogeneous population of cells [13] making up a large portion of our brain, outnumbering neurons and increasing in number and complexity with increasing phylogenetic complexity [14]. They are active participants in the regulation of neuronal homeostasis and excitability [15,16,17] as well as in mediating synaptic plasticity and network synchronization [18,19]. They play an integral role in forming and maintaining the BBB [20] and are a key downstream player in the neuroinflammatory response [21,22]. Dysregulation of any of these functions can lead to aberrant neuronal activity, predisposing to seizure generation [11,23,24]. Furthermore, reactive astrogliosis, characterized by astrocyte hypertrophy and altered gene expression [25], is characteristic for the epileptic focus [26], with changed functions of astrocytes, release of gliotransmitters, pro-inflammatory cytokines, and chemokines exerting both seizure-promoting and seizure-protective effects on the microenvironment in the brain (Figure 1). The role of astrocytes in epilepsy and their critical functions in regulating neuronal excitability were initially studied in the context of convulsive epilepsies, as extensively reviewed by our group and others [11,16,24]. Briefly, well-established astrocyte-mediated mechanisms in epileptogenesis and seizure generation include gliotransmitter release, glutamate-dependent signaling (via astroglial metabotropic and ionotropic glutamate receptors), potassium regulation through astroglial K^+^ channels (Kir 4.1), changes in gap junctions (GJs), maintenance of electrolyte balance and osmolality, BBB regulation, blood flow and energy metabolism modulation, and synapse formation support through NG2 glia production (see review [11]).

In the following paragraphs, we focus on astrocytic dysregulation in absence epilepsy, as research on the role of astrocytes in genetic generalized epilepsies with non-convulsive seizures remains limited. Emerging research on genetic rodent models, particularly those of generalized non-convulsive absence epilepsy, holds promise for advancing our understanding of glial mechanisms in epileptogenesis and ictogenesis [11,27].

### 2.2. Astrocytic Dysregulation in Absence Epilepsy

The most well-studied idiopathic generalized epilepsy in relation to astrocytic involvement is absence epilepsy, which is characterized by spontaneous spike-and-wave discharges in the EEG. In humans, paroxysmal activity in absence epilepsy is initiated in frontal and parietal cortices [27], while in rodent models, it originates in the peri-oral region of the primary somatosensory cortex and then spreads to the ventrobasal nuclei of the thalamus and the thalamic reticular nucleus [28,29]. Hyperexcitability within the thalamocortical circuit is central to the generation of spike-and-wave discharges, and increased neuronal activation in the cortex, in the form of c-fos activation, is found in rats with genetic absence epilepsy (GAERS) on postnatal day 14, before seizure onset [30], supporting cortex as the initiation zone for seizures. GAERS start displaying spontaneous seizures when they are 1 month old, which increase in frequency with age, and by 3–4 months, all animals display spike-and-wave discharges. This is followed by detection of unilateral interleukin (IL)-1beta (IL-1β) expression in the cortex of glial fibrillary acidic protein (GFAP)-expressing astrocytes on postnatal day 20, with the same animals displaying immature spike-and-wave discharges. This is supported by findings that GFAP is upregulated in the cortex prior to seizure onset [31], while IL-1β is observed bilaterally in reactive astrocytes of the somatosensory cortex, but not in adjacent cortical areas or in extra-cortical regions [30]. Furthermore, inhibition of IL-1β production in adult GAERS was able to inhibit the number of spike-and-wave discharges and duration, suggesting a role for IL-1β in absence seizure generation [30]. IL-1β, together with tumor necrosis factor-alpha (TNF-α), was also shown to be upregulated before the onset of seizures in another polygenetic rat model of absence seizures, namely the Wistar Albino Glaxo rats from Rijswijk (WAG/Rij) [32]. In the same model, treatment with the monoclonal antibody tocilizumab, which targets the IL-6 receptor, reduced the number of spike-and-wave discharges that developed during epileptogenesis. Additionally, acute application of tocilizumab after the establishment of absence epilepsy reduced both the number and duration of spike-and-wave discharges [33]. Reactive astrocytes do not only produce cytokines but change the functional profile of the astrocyte differently depending on the inducing factor [34]. How and where this occurs in absence seizure generation is not clear, but astrocyte-associated changes have been found in the thalamocortical loop, in both cortex and thalamus. The term “epileptogenesis” in the context of rat models of genetic absence epilepsy has been debated due to differences in the clinical presentation of absence seizures between humans and animal models. While human absence seizures often occur in infancy and remit with maturity, rat models develop seizures after weaning, persisting into adulthood. However, it is important to note that absence seizures can persist into adulthood in a significant number of patients (49% in [35]; 35% in [36]). This persistence highlights the relevance of studying absence seizures beyond childhood. Recently, rat models have been identified as potential models for “absence epileptogenesis”, providing critical insights into the progressive development of epileptic networks [37]. This progression is particularly evident during the “latent period” preceding seizure onset—up to P50–60 in WAG/Rij rats and P20–30 in GAERS. As highlighted by Russo et al. [37], genetic predisposition in GAERS and WAG/Rij rats acts as the initial “insult”, leading to the formation of bilateral cortical epileptic foci. These foci drive network rearrangements that ultimately result in spontaneous seizure activity. This perspective supports the use of the term “epileptogenesis” to describe the gradual development of hyperexcitable networks in genetic rat models, offering a platform for investigating early interventions, such as ethosuximide, which has demonstrated antiepileptogenic efficacy when administered during the latent period [38].

Changes in levels of extracellular gamma-aminobutyric acid (GABA) and tonic and phasic inhibition in the absence seizure circuitry are a core aspect of absence epilepsy [39,40,41,42]. Tonic inhibition is increased in thalamocortical neurons of the ventrobasal thalamic nucleus in different models of absence seizures [43], and specific activation of extrasynaptic GABA_A_ receptors was found to be necessary and sufficient to induce absence seizures in absence models and wildtype rats [44]. Increased thalamocortical inhibition in absence epilepsy was shown to be dependent on compromised GABA-transporter-1 (GAT-1) function [44], which in the ventrobasal thalamic nucleus is expressed in astrocytes [45]. In GAERS, this increase was found to have a distinct developmental onset with tonic GABAergic current in thalamocortical neurons doubling from postnatal day 16 [44]. Further supporting an increased GABA_A_ function in the generation of absence epilepsy are increased levels of the progesterone metabolite allopregnanolone at the time of seizure onset in the thalamus, cortex, and plasma of WAG/Rij rats [46]. GABA_A_ expression and opening is highly positively regulated by neurosteroid fluctuations [47,48] produced in the periphery as well as locally, by astrocytes, neurons, oligodendrocytes, and microglia [49,50,51,52]. The appearance of increased tonic inhibition in the thalamus, well before seizure onset, suggests that thalamic astrocytes play a crucial role in the development of absence epilepsy, as well as in absence seizure generation. Conversely, when labeled glutamate was infused through a microdialysis probe into the cortex of awake, unrestrained GAERS, a significant decrease in glutamate uptake was observed. This suggests that impaired astrocyte uptake may contribute to cortical hyperexcitability [53]. Further evidence of dysfunctional glutamate metabolism and reduced expression of astrocyte glutamate transporters in juvenile GAERS, as well as in primary cortical astrocyte cultures from GAERS prepared on postnatal day 0, indicates that impaired astrocyte glutamate uptake may play a role in the underlying pathology of absence seizure development [30,54]. Furthermore, metabolic studies in GAERS rats reveal enhanced metabolism in cortical astrocytes and an increased glutamine–glutamate cycle, leading to increased glutamate and glutamine levels and decreased GABA [55,56].

### 2.3. Astrocyte Contribution to Absence Ictogenesis

Astrocyte gap junction (GJ) coupling, primarily mediated by connexins (Cx) Cx30 and Cx43, ensures high intercellular connectivity within the astrocyte network, facilitating the exchange of ions and small molecules [57]. Cavdar et al. [58] showed that astrocytes in the somatosensory cortex, the ventrobasal thalamic nucleus, and thalamic reticular nucleus displayed increased expression of both Cx30 and Cx43 in GAERS and WAG/Rij compared to Wistar rats, suggesting a potential substrate for increased synchronization and its contribution to absence seizure generation [59]. The broad-spectrum GJ blocker carbenoxolone has been shown to decrease the duration of spike-and-wave discharges in GAERS rats and to reduce the amplitude and duration of epileptiform activity in thalamocortical slices [60,61]. The effects of carbenoxolone, however, vary across different absence epilepsy models [62,63], sometimes worsening absence seizures [64]. A further complicating interpretation is the direct effects of carbenoxolone on neuronal GJs, synaptic receptors, and voltage-gated calcium channels [65,66,67,68]. To get around this problem, Vincze et al. [64] used specific neuronal GJ blocker quinone together with a Cx43 antibody to confirm the results of carbenoxolone and to distinguish between neuronal and astrocyte GJ effect. Similarly, selective optogenetic activation of astrocytes in the ventrobasal thalamus revealed differential effects on absence seizures between GAERS and WAG/Rij rats [69]. In GAERS, this activation led to an increased duration of spike-and-wave discharges, whereas in WAG/Rij rats, it resulted in an increased frequency of these discharges [69]. This suggests that different mechanisms may underlie seizure generation between models or that astrocyte activation may have differential impact depending on seizure frequency. Notably, seizure frequency is higher in the WAG-Rij rats compared to GAERS, which could influence these varying responses. Activation of astrocytes has previously been shown to enhance inhibitory and excitatory neurotransmission alike, with different effects on different neurons in the circuitry [70] and different outcomes depending on whether the activation is global or localized [71,72].

In contrast, the astrocyte-modulating agent arundic acid (ONO-2506) has shown promise in experimental models. This agent selectively inhibits the production and release of S100B [73] and systemic administration was found to dose-dependently reduce the frequency and duration of absence seizures in a mouse model of absence epilepsy [74], while having no effect on induced convulsive seizures [75]. Furthermore, arundic acid increased extracellular levels of GABA and kynurenic acid, suggesting that the seizure-suppressing effect of arundic acid is partly mediated by enhanced inhibitory transmission [76].

In summary, it is still unclear if astrocytes drive the initial pathology of absence epilepsy or if pathological changes in the environment induce astrocyte reactivity, causing perpetuation of the pathological phenotype. What is clear is that astrocytes have a significant impact on epileptogenesis and ictogenesis. Continued research into astrocyte–neuron interactions, gliotransmission, and astrocyte-specific genetic changes will create avenues for novel seizure-controlling and disease-modifying strategies aimed at modulating astrocyte function.

## 3. Microglia

Microglia, as brain-resident macrophage-like cells, continuously interact with neurons and other glial cells to modulate neural activity in both physiological and pathophysiological conditions [77]. Microglia cells constitute approximately 10–20% of total glia, whereas astrocytes, oligodendrocytes, and NG2 glia account for about 80–90% of the total glial population [78,79]. Glial cells are today recognized as synaptic partners. The crosstalk among neurons, microglia, astrocytes, and oligodendrocytes through synaptic and electrical junctions has received considerable attention, and today, this concept is called penta-partite synaptic composition (Figure 1). In penta-partite composition, each element, such as microglia and/or neuron, signals and communicates with the other elements to regulate the complex structural and functional milieu of the CNS. For example, chemokine (CX3C motif) ligand 1 (CX3CL1, also known as fractalkine) is expressed by neurons, and its receptor CX3CR1 (fractalkine receptor) is mainly expressed by microglia in the brain parenchyma [80]. Therefore, neuron–astrocyte–microglia–oligodendrocyte communication is vital for the integrity of the neural circuit. Microglia cells, with highly motile and dynamic processes, consistently monitor the surrounding microenvironment and continuously interact with neurons to screen neural activity. Communication with neurons and other non-neuronal cells leads to morphological and functional changes in microglia under physiological and pathophysiological conditions. Li et al. [81] reported that neural activity attracts microglial processes and induces the formation of microglial bulbous endings, which are crucial structures for microglia to interact with neuronal structures and synapses. The neuron–microglia communication, characterized by direct interactions between microglial processes and neuronal axons and dendrites, has been well confirmed, and this specialized nanoarchitecture has been named ‘axodendritic microglial junctions’. In addition to the contacts with neuronal axons and dendrites, Cserép et al. [82] showed that microglial processes also contact soma and that microglia monitor neuronal activity through somatic microglial GJs.

Despite the accumulating knowledge on microglial properties under physiological conditions, little is known about whether and how microglia modulate the structure and function of neural circuits under pathological conditions. Microglia that quickly respond to pathogens and to initial insults, such as traumatic brain injury or stroke, contribute to neuroinflammation and/or an imbalance in the orchestration of neurotransmission, besides playing a role in synthesizing and releasing neurosteroids as an anti-epileptogenic target [83]. Therefore, microglia cells are a key homeostatic regulator in the healthy brain and also seem to be involved in the pathophysiology of brain disorders, such as epilepsy, dementia, Alzheimer’s disease, multiple sclerosis, and amyotrophic lateral sclerosis [84,85].

### 3.1. The Role of Microglia in Neuroinflammation

Neuroinflammation is, indeed, a complex response that helps to maintain homeostasis, thereby enabling the CNS to cope with epileptogenic insults. Neuroinflammation can be triggered by a variety of factors, including an insult (trauma/infection/autoimmunity/stroke) and/or genetic condition. Neuroinflammatory response involves the biosynthesis and release of molecules with inflammatory properties from neurons and brain-resident cells—chiefly, activated microglia and astrocytes. This communication between microglia and neurons is bidirectional, involving several important factors and signaling axes, including purinergic, nitric oxidergic, and adenosinergic pathways or vesicular nucleotide transporters [86]. Microglia, as one of the primary actors in the neuroinflammatory process, become activated and undergo morphological changes. Activated microglia are associated with reactive microgliosis and elevated phagocytosis, which is actively involved in maintaining tissue homeostasis with immunomodulatory effects. This is coordinated by a variety of molecules that regulate chemoattraction, engulfing, and degradation [87]. Reactive microglia release and express a wide range of pro-inflammatory mediators, including cytokines (such as IL-1, IL-6, and TNF-α), chemokines (such as CCL1, CCL5, and CXCLs), purinergic P2X7 receptors, and high-mobility group box 1 (HMGB1), which activates inflammasomes. They also release adenosine triphosphate (ATP), adenosine, glutamate transporters, prostaglandins, and nitric oxide and contribute to the generation of reactive oxygen and nitrogen species. The release of these mediators activates a series of signaling pathways and orchestrates the neuroinflammatory response [88,89]. For example, cytokines, a group of small polypeptides with a wide variety of potential actions, rapidly increase in response to injury, infection, or any insult. Therefore, cytokine levels, which play a crucial role in tissue repair during these acute pathological conditions, are usually classified as either pro-inflammatory or anti-inflammatory based on their effects in peripheral tissues. In one of the pioneer studies, Vezzani and co-workers [90] showed the involvement and release of inflammatory cytokines and molecules, leading to an increase in neuronal excitability and prolonged seizures. Another work in the context of microglia–chemokine interactions showed that CX3CL1 (fractalkine), which is produced in neurons, signals to microglial cells through its sole receptor, CX3CR1 [91]. Furthermore, the role of the microglia–chemokine interaction has been studied by using minocycline, which is considered an inhibitor of microglial activation [75,76,77]. Minocycline blocks the proliferation of microglia and the expression of CD68, a member of the lysosomal/endosomal-associated membrane glycoprotein family [91,92,93,94]. Administering minocycline 12 h before status epilepticus induced by intrahippocampal kainic acid injection reduced the number of cleaved caspase-3+ apoptotic cells and TdT-mediated dUTP nick end labeling (TUNEL)^+^ damaged cells in the CA3 and CA1 regions of the hippocampus [92]. This suggests that activated microglia may contribute to neurodegeneration following seizures. From the perspective of seizure severity, it has been demonstrated that activated microglia also play a role in the pilocarpine-induced status epilepticus (SE) model [93,94]. A two-week minocycline treatment starting one day post-SE decreased the frequency of spontaneous recurrent seizures, as well as seizure duration and severity. The inhibition of microglial activation by minocycline also provided protection against developmental seizures; however, minocycline is not specific for microglia and also affects macrophages and other immune cells, which could contribute to its observed effects [95]. In the SE that was induced by kainic acid on postnatal day 25, latency to kainic acid-induced secondary SE on postnatal day 39 was reduced; however, minocycline treatment one day after the initial SE restored latency to secondary SE [79]. These findings indicate that the initial SE primed the microglia and potentially other immune cells, leading to a lower seizure threshold within two weeks. Further, the pharmacological depletion of microglia has been shown to exacerbate seizures in a mouse model of viral infection-induced epilepsy [96], indicating that the depletion of microglia reduces the local immune response to infection and aggravates epilepsy outcomes [96,97]. These results highlight the dual role of microglia in epilepsy. While microglial activation is often associated with neuroinflammation, recent evidence suggests that microglia can also provide protective effects by managing inflammation and facilitating repair. Thus, responding to the specific context of the pathological environment, microglia exhibit dynamic and multidimensional states. Depending on circumstances, they may function as both anti-epileptic and pro-epileptic, underscoring the complexity of their role in epilepsy progression [98].

Building on the complex role of microglia in epilepsy, recent studies have further elucidated specific molecular mechanisms and pathways through which microglial activation contributes to the pathogenesis of seizures and neuroinflammation. The Audinat team showed that in kainate-induced SE, there is an involvement of purinergic P2X4R in microglial functions. They found that SE is associated with an induction of P2X4R expression in the hippocampus, mostly localized in activated microglial cells [99]. Purinergic P2X7 activity seems to be one of the main triggers of IL-1β processing and contributes to TNF-α production, leading to neuroinflammation [99]. Further, Fu et al. [100] demonstrated a higher number of Iba-1-labeled microglia and downregulation of miR-221-3p expression in the hippocampal CA1 and CA3 regions of epileptic mice compared to control animals. The intervention using an inhibitor of MiR-221-3p mimics/HIF-1α suppressed the activation of microglia and the release of inflammatory mediators, which relieved epileptic seizures in mice [100]. This outcome suggests that miR-221-3p/HIF-1α is an essential component in microglia function in the pathogenesis of epilepsy and that microRNAs are functional regulators of epileptogenesis.

In a human TLE with hippocampal sclerosis and a rat pilocarpine model of TLE, Ravizza et al. [101] reported IL-1β and IL-1 receptor type 1 broadly expressed by astrocytes, microglia, and neurons, suggesting the role of the chronic activation of the IL-1β/IL-1RI system in the onset of epileptogenesis. Furthermore, both in hippocampi from patients and in rat chronic epileptic tissue, they observed strong IL-1β and IL-1RI immunoreactivity in perivascular astrocytic end feet impinging on blood vessels and in endothelial cells of the microvasculature in brain areas exhibiting BBB damage. In another well-established rodent SE-induced TLE model developed by the administration of kainic acid, microglia exhibited increased production and secretion of inflammatory cytokines and molecules, including ILs, IL-1β, CXCL8, and TNF-α, following seizures [102]. Furthermore, experimental febrile SE in rats caused rapid and persistent increases in pro-inflammatory cytokine levels, including elevated IL1β, TNF-α, COX-2, and IL-6, but also marked the activation of prostaglandin E_2_ signaling [100]. They found that the administration of the glucocorticoid dexamethasone following febrile SE blocked microglial activation and proliferation in the hippocampus [103]. Moreover, Wyatt-Johnson et al. [104] demonstrated that the inhibition of the colony-stimulating factor 1 receptor (CSF1R; regulator of microglial survival and proliferation) by the administration of PLX3397 significantly decreased the number of microglia in the hippocampus of rats in a rat pilocarpine model of SE. Wyatt-Johnson and Brewster [105] suggested phagocytic signaling molecules as novel therapeutic targets for epilepsy. Moreover, Hughes and Appel [106] revealed that neuronal activity bidirectionally balances microglial association with neuronal cell bodies and myelin phagocytosis in the brain. From the perspective of penta-partite synapse, they suggested the crosstalk neuronal activity-regulated role of microglia in modifying developmental myelin targeting by oligodendrocytes.

In addition to being produced locally in the CNS, molecules such as IL-1β, IL-1 receptor antagonist (IL-1RA), IL-6, and TNF-α can also enter the brain from the bloodstream [107]. The levels of pro- and anti-inflammatory cytokines, including ILs and TNF-α, were measured in the blood and cerebrospinal fluid of children with febrile seizures [107]. These pro- and anti-inflammatory cytokines showed a clear increase in both blood and cerebrospinal fluid samples and were found to be associated with febrile seizures using logistic regression analysis. This suggests the involvement of both peripheral and brain cytokine networks in the pathophysiology of febrile seizures. Furthermore, microglial TNF induces the expression of vascular cell adhesion molecule-1 and intercellular adhesion molecule-1 in endothelial cells, favoring the infiltration of peripheral immune cells, which—by the production of more cytokines and chemokines—aggravates neuroinflammation [108,109]. The main peripheral immune cells that infiltrate the brain are neutrophils, monocytes/macrophages, B-cells, and T-cells. The infiltration of neutrophils is involved in the induction of acute brain inflammation after status epilepticus, and seizure frequency correlates with the number of infiltrating monocytes. On the anti-inflammatory side of the coin, microglia release different mediators (TGF-α, IL-4, and IL-10) to counteract excessive activation of the astrocytes. These cells also produce anti-inflammatory mediators (such as TGF-α, orosomucoid-2) to reduce microglial activation in epileptogenesis [109].

In focal cortical dysplasia type I and II, Iyer et al. [110] examined the inflammatory cell components and the induction of major proinflammatory pathways and molecules in surgical specimens using immunocytochemical methods. They found the prominent activation of microglia in the focal cortical dysplasia type II specimens and close association of activated immune cells with abnormal cell types, such as dysplastic neurons and balloon cells, displaying mTOR activation.

Toll-like receptor (TLR) signaling, which is activated in epilepsy, is involved in neuroinflammation and cytokine production during epileptogenesis and considered a biomarker in epilepsy models [111]. Particularly, the TLR4 signaling pathway is considered one of the important bridges connecting innate immunity and acquired immunity. TLR4 is mainly expressed in microglia and mediates microglial activation, suggesting the role of microglia in linking immunity and epilepsy. In the pilocarpine-induced rat model of epilepsy, the downregulation of the TLR4/NF-kB inflammatory pathway in epilepsy inhibited microglial activation and the expression of the inflammatory factor CD6, which reflects the strong phagocytic ability of microglia [112].

### 3.2. The Role of Microglia in Inhibitory/Excitatory Imbalance

From the perspective of the underlying factors of epileptogenesis or epilepsy, another neurobiological mechanism proposed is the neurotransmitter system and glutamatergic/GABAergic homeostasis in the CNS. It is generally accepted that glutamatergic signaling increases during epileptogenesis, while inhibitory GABAergic signaling decreases, leading to an imbalance between excitation and inhibition [113,114]. This imbalance produces the overactivation of glutamate, the burst firing of increased synchronous electrical neuronal activity, and excitotoxicity. A Ca^2+^ overload ultimately triggers intracellular Ca^2+^-dependent signaling cascades, leading to excitotoxicity [115]. This excitotoxicity, which is a cell injury mechanism, is triggered by excessive glutamate release from neurons as well as glial cells. The glutamate should be removed by Na^+^-dependent and independent glutamate transport systems, which play a critical role in glutamate uptake [116]. The emerging evidence suggests that activation of inhibitory interneurons can paradoxically contribute to epileptogenesis. GABA_A_ receptors contribute to the generation of interictal spikes and play a role in the initiation and maintenance of ictogenesis [117]. These effects are mediated, in part, by interneuron action potential firing and associated extracellular [K^+^] elevations, which can depolarize the chloride (Cl^−^) equilibrium potential and promote network hyperexcitability [117,118]. In the pilocarpine model of MTLE, activation of parvalbumin positive PV (+) interneurons—associated with an increase in inhibitory signaling—provides an overall decrease in seizure occurrence but paradoxically leads to seizure initiation once the limbic network excitability reaches a suitable condition for ictogenesis [119]. This complex interplay between inhibitory and excitatory mechanisms underscores the multifaceted contributions of interneurons to epileptiform activity and needs further investigation.

In addition to the modulatory role of synaptic GABA_A_ receptors in ictogenesis and epileptogenesis, extrasynaptic GABA_A_ receptors are sensitive to various endogenous, naturally occurring neurosteroids and synthetic neurosteroids such as allopregnanolone (also known as tetrahydroprogesterone) and ganaxolone, a synthetic analogue of the naturally occurring neurosteroid allopregnanolon. Experimental and clinical findings demonstrate the involvement of endogenous neurosteroids and their synthetic analogues as modulators of neuronal excitability in the context of epileptic disorders. Allopregnanolone and ganaxolone have been demonstrated to produce anticonvulsant activity in models of pilocarpine-induced SE [120]. Furthermore, allopregnanolone showed greater antiseizure potency in SE and kindling in mice. This anti-seizure effect of allopregnanolone was associated with higher levels of the extrasynaptic delta subunit of GABAA receptors [121]. Moreover, neurosteroids are under clinical evaluation for their potential therapeutic use in pharmacoresistant epileptic patients, as well as CNS disorders. Human microglia has been recently shown to synthesize allopregnanolone, which has numerous findings supporting it as an antiepileptogenic steroid [122]. Moreover, trilostane, as an inhibitor of 3β-hydroxysteroid dehydrogenase, was shown to delay epileptogenesis by increasing the allopregnanolone brain levels [123].

Glial and neuronal excitatory amino acid transporters (EAATs) ensure low resting neurotransmitter concentrations in the synaptic cleft and prevent glutamate excitotoxicity by transporting glutamate from the synaptic cleft into neuronal and glial cells [124]. Five high-affinity Na^+^-dependent EAATs in the CNS have been cloned as EAAT1-EAAT5. EAAT1 and EAAT2 are primarily located in glia, and EAAT3-EAAT5 are ubiquitously expressed in neurons [125]. In rodents, the EAAT1 analogue has been termed GLAST, EAAT2 as GLT-1, and EAAT3 as EAAC1 [126]. The EAAT1 and EAAT2 (GLAST and GLT-1 in rodents), which are expressed in astrocytes, mediate glutamate uptake to provide rapid clearance of glutamate and prevent glutamate-mediated neuronal injury [127]. The EAAT1/EAAT2 in human astrocytes and GLAST/GLT-1 in rodents potentially influence crosstalk between glutamatergic and GABAergic neurotransmission [128]. In addition to secondary active glutamate transport, EAATs and GLAST/GLT-1 also serve a dual function as glutamate-gated anion channels, which are also involved in the regulation of internal Cl^−^ concentrations [129]. This dual function allows cells to respond to glutamate and to provide a feedback signal about the concentration of glutamate released into the synaptic cleft and also plays a role in the Cl^−^ homeostasis. Cl^−^ channels expressed in microglia are categorized as members of volume-regulated Cl^−^ channels (VRCCs) and Cl^−^ intracellular channels (CLICs). These channels are involved in numerous steps in the microglial activation process, such as the chemotaxis, ramification, proliferation, and production of pro-inflammatory cytokines and neurotoxic factors, as well as phagocytosis related to neurodegenerative diseases [130,131]. Preclinical studies demonstrate that Cl^−^ channel blockers like 5-nitro-2-(3-phenylpropylamino) benzoic acid (NPPB) and IAA94 attenuate neuroinflammation and oxidative stress in animal models of neurodegenerative diseases [130,132,133].

In glial cells, glutamate transporters functioning as anion channels have been shown to contribute to glial chloride homeostasis [129]. To prevent excitotoxic effects, the astroglia glutamate transporters GLT-1 and GLAST are of vital importance in maintaining the glutamatergic/GABAergic and Cl^−^ homeostasis. Furthermore, K^+^ channels play important roles in microglia functions and thus constitute potential targets for the treatment of acquired epilepsies and for the regulation of Ca^2+^ signaling in microglia by a selected number of ion channels and receptors [134].

Accumulating evidence also suggests that glutamate and its transporters have a functional role in immunoregulation that is well beyond its role as a neurotransmitter and homeostasis in the brain. Furthermore, the Na^+^-independent glutamate transport system is typically a Cl^−^-dependent glutamate/cystine antiporter, which exchanges internal glutamate for cystine, the oxidized form of cysteine [127]. This transport system has been termed system X_AG_ and system X_c_ [135]. In addition to astrocytes, in which glutamate is transformed into glutamine to transport back to neurons, microglia have two systems for glutamate transport: GLT-1 for transport into the cells and the X_c_ system for transport out of the cells [136]. The expression of GLT-1 protein in activated microglial cells suggests a neuroprotective role of microglia against glutamate excitotoxicity following neuronal traumatic injury [137]. Persson and Rönnbäck [127] demonstrated that microglia in cultures express GLT-1, but not GLAST, and transport glutamate from the extracellular space. They also showed that TNF-α can induce increased microglial GLT-1 expression, possibly by associating the expression with inflammatory systems. Despite the importance of the balance between excitatory and inhibitory synaptic function in epilepsy, few studies have examined microglial regulation, and the reality remains elusive from the perspective of epileptogenesis [138]. The Biber team [139] examined the effects of NMDA-induced excitotoxicity on neuronal degeneration and microglial activation in mouse organotypic hippocampal slice cultures. They showed that microglia replenishment rescues neurons from excitotoxicity.

Dutuit et al. [54] demonstrated decreased expression of glutamate transporters in rats with genetic absence epilepsy before seizure occurrence. Ingram et al. [140] showed substantial and significant increases in GLT-1 mRNA levels in the ventromedial nucleus of the thalamus and the subthalamic nucleus of GAERS. The increases in GLAST mRNA were found in the primary somatosensory cortex and temporal cortex of GAERS. These data, along with previous studies, suggest that regional imbalances in GABAergic and glutamatergic systems may be associated with the pathogenesis of absence seizures in genetic absence epilepsy. Features that ensure brain homeostasis—such as ionic composition, volume regulation, neurotransmitter elimination, neuroglial connectivity, gliotransmission, Ca^2+^ signaling, and cytokine release—are fundamental elements of glial function, and there is a need for future studies addressing these aspects in the context of genetic generalized epilepsies [141]. 

However, this longstanding proposed mechanism of ictogenesis and epileptogenesis seems to be incomplete. At least, it is evident that such a stereotype does not apply to various forms of epilepsy, including generalized epilepsies characterized by non-convulsive seizures. It has been shown that there is an excess of GABAergic inhibition in the thalamic neurons and, in contrast, an imbalance between inhibitory and excitatory neurotransmission in the hyperexcitable cortex at the regional level. Therefore, when considering the mechanisms, it would be a more accurate approach to address the subtypes of epilepsy individually. However, most of the recent evidence focuses on glial contribution to convulsive epileptic activity such as TLE. Although neurons and astrocytes are known as the primary mediators in the process of neuroinflammation in TLE, microglia, the unique resident immune cells of the brain, also play a critical role in the pathogenesis of epilepsy with different etiologies.

In summary, evidence provides strong support for the microglial contribution to the epileptogenic networks through a series of neuroinflammatory short- and long-term signaling after an insult goes beyond inflammation. Here, in addition to neuroinflammation, we addressed the role of the infiltration of the peripheral immune system and the structural and functional collapse of the balance between excitatory and inhibitory neurotransmission (Figure 1). All this evidence reveals the complex nature of epileptogenesis and indicates the need for new perspectives on penta-partite synaptic organization. Thus, the necessity of such an approach becomes particularly clear for drug-resistant epilepsy patient groups. Novel therapeutic and modulatory approaches should target this complex orchestration.

## 4. Oligodendrocytes

Oligodendrocytes, specialized glial cells in the CNS responsible for supporting and maintaining neurons, have become recognized as part of the functionally integrated neuron–glia network in seizure pathogenesis and epilepsy. The main function of oligodendrocytes, which are connected to other glial cells through GJs, is to form the myelin sheath that insulates axons, enabling faster and more efficient neurotransmission.

**Figure 2:**
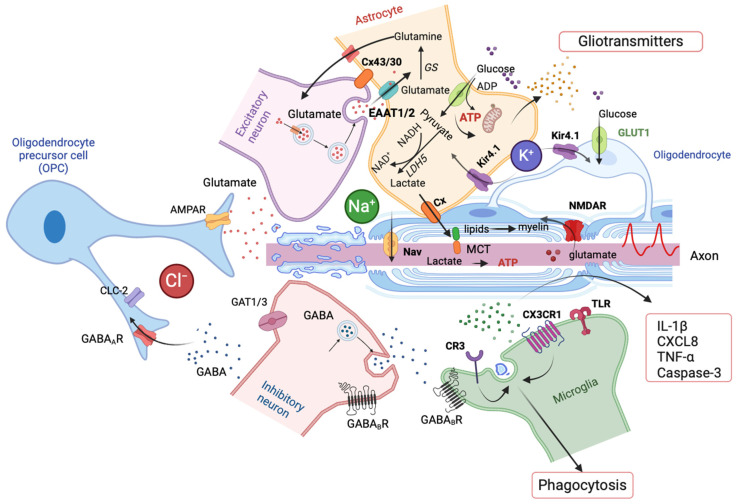
Neuron–glia crosstalk in penta-partide synaptic composition. The schematic illustrates the complex interactions between neurons and glial cells (astrocytes, microglia, oligodendrocytes, and oligodendrocyte precursor cells (OPCs)) that contribute to epileptogenesis. It highlights key pathways and molecular mechanisms within a neuroinflammatory microenvironment, emphasizing the role of ion homeostasis (e.g., K^+^, Na^+^, Cl^−^), gliotransmitter release from reactive astrocytes, and excitatory and inhibitory synaptic signaling. Accordingly, the changes in glial membrane expression and trafficking of inwardly rectifying potassium (K^+^) channel 4.1 (Kir4.1), excitatory amino acid transporter (EAAT1/2), and the GJ proteins connexin 43/30 (Cx43/30) results in an increase in extracellular concentrations of K^+^ and glutamate, which promotes network hyperexcitability and seizures. The figure also explores metabolic support, oligodendrocyte involvement in myelin and lipid transfer, and direct interactions of OPCs with both excitatory and inhibitory neurons. Additionally, microglia are depicted in their roles in cytokine release and phagocytosis. Created in BioRender 2024 (https://BioRender.com/c12o944, accessed on 10 November 2024).

Myelin, the multilayered lipid-rich membrane formed by oligodendrocytes around neuronal axons, is essential for fast and efficient action potential propagation in the CNS. It acts as a natural electrical insulator for axons, decreases membrane capacitance, and is a vital structural component in regulating precise synaptic interactions between neurons [142]. In contrast to Schwann cells that form a single myelin sheath around axons in the periphery, each oligodendrocyte generates multiple layers of myelin (between 20 and 50 myelin sheaths), allowing for rapid conduction of action potentials between brain regions [143]. OPCs promote myelin formation by differentiating into myelinating oligodendrocytes during the late prenatal and postnatal stages of development [144]. OPCs exhibit immunoreactivity to neuron-glial antigen-2 (NG2) proteoglycan; therefore, they have also been widely known as NG2 glia, the fourth type of glial cells in the CNS [145]. During development, oligodendrogenesis and myelination are regulated by a combination of cell-intrinsic and dynamic external microenvironmental signals such as neuronal activity, vasculature, astrocytes, and microglia [146,147]. Under conditions involving myelin disruption, including some forms of epilepsy [148,149], OPCs rapidly proliferate, migrate to affected areas, and differentiate into mature oligodendrocytes to re-wrap exposed axons, thereby restoring the myelin sheath [150]. This endogenous repair process, known as “remyelination”, is primarily driven by mature oligodendrocytes and OPCs. In conditions where OPC proliferation or differentiation is impaired, there may be insufficient remyelination following neuronal injury or stress [151]. This can leave demyelinated axons vulnerable to hyperexcitability and enhance susceptibility to seizures, suggesting that myelin sheath, mature oligodendrocytes, and OPCs are most likely involved in epileptogenesis. Moreover, OPCs receive direct inputs from both glutamatergic excitatory neurons and GABAergic inhibitory interneurons, which are crucial for maintaining the excitation/inhibition balance in neuronal networks [152]. These interactions enable OPCs to sense and respond to neuronal activity, thereby actively participating in modulating neural circuits and network dynamics [8]. Additionally, OPCs play a role in maintaining network homeostasis by regulating extracellular ion concentrations and responding to injury or disease. They can secrete signaling molecules that influence inflammation, repair, and neuroplasticity. These functions are independent of their differentiation into oligodendrocytes, justifying their inclusion as distinct entities within the penta-partite synaptic model [9] (Figure 2).

Cl^−^ homeostasis plays a vital role in the proliferation and development of oligodendrocytes [153,154]. The voltage-gated Cl^−^ channel (CLC) has been identified as a positive modulator of OPC differentiation and myelination [155]. Specifically, CLC-2 is expressed in both OPCs and mature oligodendrocytes. In vitro studies have demonstrated that pharmacological inhibition of CLC-2 currents using GaTX2, a specific blocker of CLC-2, has a more pronounced effect in OPCs compared to oligondendrocytes [155]. This finding suggests that CLC-2 plays a crucial role in facilitating the differentiation of OPCs into fully developed oligodendrocytes. Disruption of CLC-2 function leads to severe and widespread vacuolation of white matter in the brains of CLC-2 knockout (KO) mice. This pathology is attributed to disturbances in ion homeostasis within the oligodendrocytic/astrocytic network, although no differences in seizure thresholds were observed among wild-type, heterozygous, and homozygous CLC-2 KO mice [156].

Recent findings offer valuable insights into the interaction between oligodendrocytes and PV (+) interneurons, which comprise half of the interneuron population in the neocortex. Interestingly, in a genetic model in which OPCs lack the γ2 GABA_A_ receptor subunit, fast-spiking PV (+) interneuron axons in the neocortex are aberrantly myelinated, and feedforward inhibition is impaired in cortical layer IV neurons, leading to excitation–inhibition imbalance [157]. A recent study demonstrated that photostimulation of OPCs induces vesicular GABA release, resulting in both phasic and tonic inhibition of nearby interneurons, while sparing pyramidal neurons in the adult hippocampal CA1 region [158]. These studies suggest that OPCs, through the formation of postsynaptic and/or potential presynaptic connections with neurons, or even via direct physical contact, play a role in modulating interneuron activity and inhibitory circuits in the brain [159]. Similarly, loss of oligodendrocyte ErbB receptor signaling causes hypomyelination, reduces the density of PV (+) interneurons, and alters the excitatory/inhibitory balance in the auditory cortex [160]. Further studies are essential to explore the emerging roles of OPCs in regulating brain function under both physiological and pathological conditions. Understanding the cellular and molecular mechanisms through which OPCs impact neural activity offers significant potential for discovering new therapeutic targets for central nervous system disorders, particularly in the context of epilepsy.

Myelination is not a static process; after an initial phase of intrinsic myelination, external experience-based modifications occur to adjust neuronal conduction and fine-tune neural pathways. This form of plasticity, known as “adaptive myelination”, is driven by both the remodeling of pre-existing oligodendrocytes and the formation of new myelinating oligodendrocytes (de novo myelination) [143,161]. Adaptive myelination plays a crucial role in cognitive functions, emotional regulation, and motor learning and is shaped by experience and lifestyle. Therefore, changes in myelination can alter neuronal dynamics and function [162]. Myelinated axons are not uniformly covered by myelin; the axon initial segment and the nodes of Ranvier remain unmyelinated and serve as critical domains for regulating axonal excitability. Abnormal oligodendrocyte function may impair this adaptive myelination, preventing proper regulation of network synchrony. This may lead to desynchronization or hypersynchronization of neuronal networks, both of which are associated with seizure generation.

Oligodendrocytes not only form the myelin sheath around axons and regulate the speed of action potential conduction, but also provide metabolic support to axons, thereby playing a critical role in fine-tuning neural circuit dynamics [162]. Oligodendrocytes have been proposed to provide axons with lactate, a metabolite that can be converted into ATP within the axons [163,164,165]. This is crucial for sustaining repetitive action potential firing [166] and preserving axonal health [167]. Impaired oligodendrocyte function may compromise the axonal energy supply, as ATP shortages can disrupt ion pump functions, leading to intracellular Na^+^ and Ca^2+^ buildup, potentially triggering neuronal hyperexcitability and seizures. Additionally, oligodendrocytes express functional NMDA receptors [168], which play a role in supporting axonal energy metabolism. These oligodendroglial NMDA receptors regulate the expression of the glucose transporter (GLUT1) and enhance glucose uptake, the precursor of lactate production [165]. Dysfunction in these NMDA receptors can reduce glucose uptake, leading to energy deficits in neurons and impaired synaptic function. This metabolic insufficiency may exacerbate neuronal hyperexcitability, contributing to the generation of epileptic seizures (Figure 2).

### 4.1. Myelin Abnormalities in Epilepsy: Evidence from Preclinical Models and Clinical Studies

Myelination involves more than just sheathing the axon; neuron axons undergo significant changes both before and during myelination. These changes include the organization and positioning of key voltage-gated ion channels, which seems to play a critical role in epilepsy. Myelin abnormalities have been previously reported in epilepsy patients, and seizures are frequently observed in demyelinating diseases such as multiple sclerosis, suggesting the potential role of myelination in promoting seizure activity [169]. Myelin and oligodendrocytes support neuronal glutamatergic transmission via the expression of glutamine synthetase (GS) and play a crucial role in maintaining K^+^ homeostasis [170]. The inwardly rectifying K^+^ (Kir) 4.1 channels found on both astrocytes and oligodendrocytes are essential for buffering excess extracellular K^+^ released during neuronal activity [171] (Figure 2). It has been hypothesized that changes in the extracellular K^+^ may be amplified by demyelinated/unmyelinated axons, resulting in hyperexcitability and seizures [169]. Numerous experimental and clinical studies have reported a link between myelin content, abnormal myelination, and epileptogenesis. Myelin abnormalities have been reported in animal models of epilepsy. Moreover, seizures have been reported in genetic and pharmacological models of demyelination [169,172]. Considering the wide range of mechanisms involved in different forms of epilepsy, it is likely that the role of myelination may also differ across these conditions. In the following paragraphs, we explore the current understanding of myelination in focal and generalized epilepsy models, highlighting how these processes differ and their implications for seizure generation and propagation. Additionally, we discuss experimental findings from both focal and generalized epilepsy models, including their relevance to therapeutic strategies targeting myelination. The findings on the role of oligodendrocytes in TLE and generalized epilepsy models are summarized below and presented in Table 1.

### 4.2. Myelin Abnormalities in Focal Epilepsies

Focal epilepsies, both human and experimental, are associated with demyelination in the white matter and reduction of oligodendrocytes, suggesting that the loss of axonal myelin may contribute to cortical hyperexcitability, ultimately leading to seizures [169]. In TLE models, such as lithium-pilocarpine-, kainic acid-, and pentylenetetrazol (PTZ)-induced kindling, loss of myelinated nerve fibers in the hippocampus, thalamus, and cortex has been associated with seizure frequency [173,174,175]. In the rat hippocampus, myelin and oligodendrocyte loss began during the acute phase of lithium-pilocarpine-induced epileptogenesis, worsening progressively through the latent and chronic phases [176]. This seizure-induced myelin damage was accompanied by activation and increased populations of OPCs throughout the acute, latent, and chronic phases, indicating that an endogenous repair process regulated by the transcription factors is initiated during epileptogenesis to compensate for the loss of myelin [176]. In animal models, treatment with the immunomodulator drugs glatiramer acetate and fingolimod resulted in a significant reduction in both demyelination and seizure frequency [174,175]. Besides, the plasticity of the developing brain has been considered a factor in seizure-induced demyelination. The immature brain displayed better tolerance to demyelination induced by seizures than the adult brain and could maintain the myelination process throughout epileptogenesis [177].

### 4.3. Myelin Abnormalities in Genetic Generalized Epilepsies

Genetic generalized epilepsy models have revealed intriguing findings related to this topic. Proteomics data reveal that myelin basic protein levels, the main components of the myelin membrane, were upregulated in the thalamus of a rat model of absence epilepsy [178]. Similarly, recent research using two different models of genetic generalized epilepsy with absence seizures (WAG/Rij rats and Scn8a^+/mut^ mice, a mutant model harboring a loss-of-function SCN8A mutation) showed that oligodendrogenesis and myelination specifically increased within the thalamo-cortical network involved in seizure generation following seizure onset [179]. Increased myelination did not occur when seizures were pharmacologically treated with the anti-absence drug ethosuximide. Interestingly, blocking activity-dependent myelination pharmacologically and genetically suppressed seizure frequency associated with a decrease in myelination [179]. In contrast to the aberrant myelination that develops after seizure onset in the genetic rat model of absence epilepsy, abnormal myelination has been detected early in life in the FAST-kindling rat strain, which is highly susceptible to epilepsy induced by amygdala kindling. In this model, myelin sheath thickness in the corpus callosum was greater than in controls, even without kindling, suggesting that altered myelination prior to seizure onset may contribute to increased seizure susceptibility [180]. These findings suggest that the abnormal or maladaptive myelination found exclusively in the epileptic seizure network may enhance seizure activity by encouraging pathological, hypersynchronous brain states [181] and shift the attention toward the epileptic networks rather than just the previously documented reduction of myelin in focal epilepsy models. Overall, these findings imply that myelination patterns may vary among different types of epilepsy, reflecting the diverse nature of this category of neurological disorders.

Building on preclinical data, the broader implications of these findings for human pathology are crucial and have yet to be fully understood. Human studies primarily utilizing magnetic resonance imaging and neuropathological analysis have reported the presence of myelin alterations in pathological conditions associated with TLE [182]. The myelin content has been investigated in post-operative brain tissue from patients with intractable epilepsy. Hu et al. [148] discovered that the levels of myelin basic protein were significantly lower in epileptic tissue compared to normal brain tissue. Diffusion tensor imaging revealed myelin loss in TLE patients, especially in the hippocampus and temporal lobe regions [183]. In genetic generalized epilepsies, frontal myelin content was found to be significantly lower in children with childhood absence epilepsy [133]. Results also showed white matter integrity impairment in the basal ganglia–thalamocortical circuit of drug-naïve childhood absence epilepsy patients [184]. Given the diversity of epilepsy syndromes and the varying roles that myelination may play in each, accelerated myelination has been linked to conditions such as focal cortical dysplasia [185] and early postnatal epilepsies [186].

### 4.4. Microenvironmental Influences on Oligodendrocyte Function

Much remains to be learned about the role of myelinating oligodendrocytes and their precursors in epilepsy and whether and how myelinating oligodendrocytes interact with other cell types in the neural microenvironment and influence myelination. Astrocytes and microglia, the other glial cells in the brain, play crucial roles in regulating myelination within the neural microenvironment. Astrocytes, another class of glia, also play a crucial role in regulating myelination. They likely support the survival and proliferation of OPCs by supplying soluble factors, such as platelet-derived growth factor and fibroblast growth factor 2 [187]. Additionally, astrocytes regulate myelin thickness as well as nodal gap length by exocytosis of thrombin protease inhibitors at the node of Ranvier [188]. Disruption of GJs between astrocytes and oligodendrocytes by the loss of connexins Cx47 and Cx30 causes myelin deficits, including vacuole formation and thinner myelin [189]. Furthermore, oligodendrocytes require external lipids for myelin production, which are supplied by astrocytes [190]. Microglia are essential for oligodendrogenesis and myelin development because they secrete several trophic factors and other signaling molecules, including chemokines and cytokines, as well as and control iron stability for OPCs [191]. Molecules released by microglia can also be inflammatory or cytotoxic to oligodendrocytes through pro-inflammatory cytokines, proteinases, and reactive oxygen species. During development, microglia also phagocytose excess myelin sheaths, a process that is reduced by increased neuronal activity [103]. Impaired microglial phagocytosis of excess myelin has been shown to increase seizure susceptibility [192], highlighting the crucial role of microglia in maintaining proper myelin formation for healthy brain function (Figure 2).

In summary, oligodendrocytes play a critical role in epileptogenesis with factors such as demyelination, disruption of axonal energy support, dysfunction of oligodendrocytic NMDA receptors, impaired K^+^ buffering, OPC proliferation and differentiation, and disrupted cellular interaction, all potentially contributing to seizure generation and the progression of epilepsy.

## 5. Concluding Remarks

The understanding of epilepsy/epileptogenesis and its therapy has traditionally focused on neuronal and synaptic mechanisms—such as neuronal excitability, neurotransmitter release, synaptic connectivity, and inhibitory and excitatory neurotransmission—that influence network excitability and seizure susceptibility [193]. However, rapidly growing scientific evidence, as discussed above, suggests that abnormal neuron–glia crosstalk within the neuroinflammatory microenvironment also plays a significant role in epilepsy pathophysiology. Dysregulation of glial functions in epileptic tissue depending on the type of epilepsy can occur via diverse mechanisms that may promote neuronal hyperexcitability, reorganization of neuronal networks, and epileptogenicity. Understanding the crosstalk among these cells not only elucidates the complex mechanisms underlying epilepsy but also highlights their potential as therapeutic targets. However, the development of glia-focused therapies for epilepsy presents several significant challenges. The complexity of glial functions, encompassing diverse roles depending on circuitry and disease state, complicates the targeting of specific functions without unintended effects on others. Moreover, the long-term effects of glia-focused therapies remain largely unknown. Chronic modulation of glial cells could lead to unforeseen impacts on brain health and function, necessitating thorough long-term studies. Addressing these challenges requires continued research and innovative approaches to developing safe and effective glia-focused therapies for epilepsy. By overcoming these hurdles, we can pave the way for novel treatments that improve outcomes for individuals with epilepsy.

## Figures and Tables

**Table 1:** Summary of findings related to the role of oligodendrocytes in TLE and generalized epilepsy models.

Aspect	TLE Models	Generalized Epilepsy Models
Animal Models	Lithium-pilocarpine, kainic acid, pentylenetetrazol (PTZ)-induced kindling.	WAG/Rij rats, Scn8a^+/mut^ mice (loss-of-function SCN8A mutation).
Myelination Changes	Loss of myelinated nerve fibers in hippo campus, thalamus, and cortex associated with seizure frequency. Endogenous repair processes, such as OPC activation and proliferation, during epileptogenesis.	Increased oligodendrogenesis and myelination in thalamo-cortical networks following seizure onset. Aberrant myelination observed early in life in certain genetic models.
Developmental Influence	Immature brains show better tolerance to seizure-induced demyelination and maintain myelination during epileptogenesis.	Abnormal myelination early in life may contribute to increased seizure susceptibility (e.g., FAST-kindling rat strain).
Pharmacological Effects	Treatment with immunomodulators (e.g., glatiramer acetate, fingolimod) reduces demyelination and seizure frequency.	Ethosuximide prevents increased myelination in absence epilepsy models. Blocking activity-dependent myelination suppresses seizure frequency and reduces myelination.
Key Insights	Myelin damage and repair are key features of focal epilepsy. Seizure-induced demyelination can be progressive.	Aberrant or maladaptive myelination enhances seizure activity by promoting pathological, hypersynchronous brain states.

## Data Availability

No new data were created or analyzed in this study. Data sharing is not applicable to this article.

## Abbreviations

PAMPs: pathogen-associated molecular patterns; DAMPs: damage-associated molecular patterns; GABA: gamma-aminobutyric acid; IL-1β interleukin-1 beta; CXCL8: C-X-C motif chemokine ligand 8 (also known as interleukin-8 or IL-8); CX3CR1: C-X3-C motif chemokine receptor 1; TLR: toll-like receptor; TNF-a: tumor necrosis factor alpha; iNOS: inducible nitric oxide synthase; IL-6: interleukin-6; NMDAR: N-methyl-D-aspartate receptor; GLUT-1: glucose transporter type 1; GS: glutamine synthetase; ATP: adenosine triphosphate; ADP: adenosine diphosphate; LDH5: lactate dehydrogenase 5; NADH: nicotinamide adenine dinucleotide; NAD: nicotinamide adenine dinucleotide; MCT: monocarboxylate transporter; Nav: voltage-gated sodium channel; CR3: complement receptor 3 (macrophage-1 antigen, or CD11b/CD18); P2R: P2 type purinergic receptor; GJs: gap junctions.
